# Pedophile, Child Lover, or Minor-Attracted Person? Attitudes Toward Labels Among People Who are Sexually Attracted to Children

**DOI:** 10.1007/s10508-022-02331-6

**Published:** 2022-09-29

**Authors:** Sara Jahnke, Nicholas Blagden, Laura Hill

**Affiliations:** 1 Department of Health Promotion and Development, University of Bergen, Postboks 7807, 5020 Bergen, Norway; 2 Department of Psychology, Åbo Akademi University, Turku, Finland; 3 Department of Psychology, Nottingham Trent University, Nottingham, UK

**Keywords:** Pedophilia, Hebephilia, Labeling, Identity, Mixed-method, DSM-5

## Abstract

The primary label for people who are sexually attracted to children (“pedophile”) is conflated with sexual offending behavior and tainted with stigma. In the present pre-registered mixed-method study, we therefore investigated attitudes and preferences regarding "pedophile/hebephile" and other labels among 286 people who report a stronger or equally strong sexual attraction to prepubescent and pubescent children than to adults. Overall, quantitative data showed acceptance of “pedophile/hebephile” as well as a range of alternative labels in a personal (*Labeling Oneself*) and a professional context (*Being Labeled by Others*). “Minor-attracted person” and “pedophile/hebephile” received generally higher support than other terms and appeared to be least divisive across three major online fora. Qualitative data revealed four themes: “Contested self-labels,” “Person-first language and pathologizing sexuality/identity,” “Stigma and shame,” and “Reclaiming the pedophile label.” Our results allow deeper insight into reasons for adopting certain labels over others, as well as difficulties of finding a non-stigmatizing label. We discuss limitations of the study and practical implications for clinical and research contexts.

## Introduction

By allowing to convey a large amount of information without using many words, the use of labels helps to make communication more efficient. This is particularly important in professional contexts, such as mental health services or science. Diagnostic labels, for example, allow clinicians to describe their patients’ symptoms, while also conveying information about the expected course of the illness as well as potential causes and interventions (Corrigan, 2007). Yet, labels may also carry negative connotations (Mendelsohn et al., 2020), contribute to public stigma (Granello & Gibbs, 2016), or be experienced as derogatory or ill-fitting (Dunn & Andrews, 2015).

The present article focuses on label preferences among people who experience a sexual attraction to prepubescent or pubescent children (i.e., pedophilia or hebephilia, respectively, according to psychiatric conventions, American Psychiatric Association, 2013; Blanchard et al., 2008).^1^ As with homo-, bi-, or heterosexuality, previous research indicates that sexual attraction to children is mostly stable throughout life (Grundmann et al., 2016; Seto, 2012, 2017), at least in cases where the attraction to children is more exclusive (Tozdan & Briken, 2019). The most common label to refer to people who are sexually attracted to children (i.e., “pedophile”) is colloquially used as a slur and/or way to refer to people who have committed sexual offenses against children (Feelgood & Hoyer, 2008; Harper & Hogue, 2015; McCartan, 2010). Previous research indicates that people who are sexually attracted to children vary in how they self-identify and navigate the stigma associated with their attraction. These studies are mostly qualitative in nature and tend to include small and/or highly selected samples of people who are sexually attracted to children (Freimond, 2013; Walker, 2020; cf. Martijn et al., 2020 who conducted a quantitative survey among users of multiple online fora).

The present survey collected information from a large multi-fora online sample of people who are sexually attracted to children. Combining the strengths of qualitative and quantitative approaches in a mixed-method design, our data permit a comprehensive look at the level of support that various labels receive in a public (i.e., academic or journalistic) or self-labeling context, as well as capturing why participants come to adopt or reject certain labels. Our findings are intended to add to our still limited understanding of how people who are sexually attracted to children manage their “spoiled identities” (Goffman, 1963) through adopting, rejecting, or reclaiming various group labels, including “pedophile/hebephile,” “boy/girl/child lover,” “person with pedophilia/hebephilia,” “pedophilic/hebephilic person,” or “minor-attracted person.”

### The Importance and Function of Labels

Over decades, social psychologists have studied the effects of social categorization on intergroup attitudes and behaviors. Labels and their connotations can activate stereotypes, attitudes, and emotions, which has implications for social judgments, empathy, and intergroup behavior (see Kawakami et al., 2017 for an integrative discussion of the consequences of social categorization). Despite common misperceptions, people who are sexually attracted to children can live offense-free (Cantor, 2014; Cantor & McPhail, 2016), and more than half of the people who commit sexual offenses against children are primarily sexually attracted to adults (Schmidt et al., 2013; Seto, 2018). Nevertheless, people who are sexually attracted to children face a harsh stigma, even when they abide by the law (Jahnke et al., 2015a, 2015b; Lehmann et al., 2020). Although “pedophilia” in a strict sense only denotes a sexual attraction to prepubescent children, it is often used synonymously with offending behavior in the media (Harper & Hogue, 2015; McCartan, 2010; Stelzmann et al., 2020) and the scientific literature (Feelgood & Hoyer, 2008). This may explain why public reactions tend to be more punitive when the “pedophile” label is used (as opposed to “people with a sexual interest in children”; Imhoff, 2015; Imhoff & Jahnke, 2018). Yet, it is not clear how alternative labels would be received by the general public.

Group labels also help individuals understand their role in society. Hence, identification with a label is assumed to affect self-esteem, depending on whether they are a member of a valued or de-valued social group (Galinsky et al., 2003; Tajfel & Turner, 1986). Yet, people who adopt a socially devalued identity are not necessarily at risk of internalizing society’s negative and stereotypical attitudes about themselves (Corrigan & Watson, 2002). There is some evidence that by reclaiming and reframing devalued labels, social groups can challenge previously stigmatized labels and protect their self-esteem (Galinsky et al., 2013; Wang et al., 2017). Deliberately reclaiming a label can infuse the label with positive connotations, foster a sense of agency and collective self-esteem, and limit the power of a majority group to use the label as a weapon against a member of the stigmatized group (Galinsky et al., 2003). In special cases (also referred to as *insular reclamation*), speakers use slurs to foster camaraderie based on their shared experience of discrimination and do not accept being labeled thusly by members of the outgroup (Jeshion, 2020). Hence, there may be differences between what people find acceptable when they label themselves or their group compared to when they are labeled by others who are not necessarily ingroup members (e.g., researchers or journalists). Groups may also decide to reject negative labels completely and instead focus on creating and popularizing their own language and terms to refer to themselves. We can detect instances of both strategies among people who are sexually attracted to children: while the patient-advocacy group B4U-ACT (2020) prefers to refer to their group as “minor-attracted persons,” others such as the “Virtuous Pedophiles” support group use the term “pedophile” self-referentially, while at the same time distancing themselves from criminal connotations (Nielsen et al., 2022).

### The Debate on Bias-Free Language

Recent decades have seen a growing interest in and intense lobbying around the correct (i.e., most respectful and least biased) way of referring to minority groups in academia and beyond (Bailey, 2019; Dyck & Russell, 2020; Williamson et al., 2020). This has led to the development of person-first and identity-first language (Dunn & Andrews, 2015). Person-first language is a type of label in which a noun referring to a person or group precedes an attribute (e.g., “a person with depression”), while identity-first language places the adjective before the personhood-noun (e.g., “a depressed person”). Emphasizing someone’s personhood as opposed to referring to a group solely by one diagnosis or condition (as in “the depressed,” “the disabled”) is believed to decrease stigma. Indeed, some experimental studies have demonstrated a stigma-reducing effect of first-person language (e.g., for “people with a mental illness” versus “the mentally ill”; Granello & Gibbs, 2016; for “(juvenile) sex offender” versus more neutral descriptions like “people who have committed crimes of a sexual nature”; Harris & Socia, 2016).

In spite of this, person-first language has garnered criticism by some disability groups or advocates who argued that this practice may inadvertently increase stigma (Dunn & Andrews, 2015). For instance, one scholar reports that person-first language is used more frequently to refer to children with than children without disabilities, and even more frequently for children with the most stigmatized conditions (e.g., intellectual disabilities; Gernsbacher, 2017). In its present edition, the publication manual of the American Psychological Association (2019) acknowledges both person-first and identity-first language as acceptable choices and places the onus on the researcher to learn about what type of language is preferred by the group that this person wants to study. This may also include the use of terms that are not based on prior expert consensus but arise from within the communities themselves.

### Labels for People Who Are Sexually Attracted to Children and Their Use in Academic Writing

A brief search on the scientific database *webofscience* (see Table 1) focusing on titles and author keywords only reveals that a great majority of publications uses the terms “pedophile,” “hebephile,” or “pedohebephile” (e.g., Beier et al., 2009; Cantor & Blanchard, 2012; Harper et al., 2016). Since about the early 2000s, person-first (e.g., “nonoffending individuals with pedophilia”; Heasman & Foreman, 2019) and identity-first (e.g., “pedophilic patients”; Berner & Preuss, 2002) labels have been used more frequently, and often in the context of offense prevention, stigma, and clinical treatment (e.g., in Jahnke et al., 2015a, 2015b; Martijn et al., 2020). Since 2017, we see a fast growing number of articles about “minor-attracted people” (e.g., Cohen et al., 2020; Grady & Levenson, 2021; Walker, 2020). Some researchers have adopted this term in their scientific writing, either to replace the terms “pedophilia” and “hebephilia” or as a catch-all for people who feel sexually attracted to minors (typically aged 17 and younger), including those in late stages of puberty or after puberty. Many of these articles focus on various experiences of community people who are sexually attracted to children (e.g., stigma, obstacles to help-seeking) as well as child sexual abuse prevention. The term “boy/girl/child lover” appeared least frequently (i.e., 5 hits), and its usage was typically limited to authors who defend adult-child sex (e.g., Brongersma, 1991). Nevertheless, even when taken together and even when limiting the search to articles published since the year 2000, the four alternative labels appear considerably less frequent than the traditional clinical terms “pedophile,” “hebephile,” or their variants.

**Table 1:** Results of database title searches in WebofScience

Label	Search string	Hits (hits after deletion of false hits)	Earliest mention
Pedophile/ Hebephile/ Pedohebephile	pedophile* OR paedophile* OR hebephile* OR pedohebephile*	322 (263 ^a^)	1964 (174 published after year 2000)
Person first label	(“person with” OR "people with" OR "individual* with" OR "participant* with" OR "men with" OR "man with" OR "woman with" OR "women with") AND (pedophil* OR paedophil* OR hebephil* OR pedohebephil*)	23 (22 ^b^)	1993 (but note that all subsequent mentions have been published after 2000)
Identity first label	(pedophilic OR paedophilic OR hebephilic OR pedohebephilic) AND (person* OR people OR individual* OR participant* OR men OR man OR woman OR women OR patient) NOT (“person with” OR "people with" OR "individual* with" OR "participant* with" OR "men with" OR "man with" OR "woman with" OR "women with" OR "patient with") AND (pedophil* OR paedophil* OR hebephil* OR pedohebephil*)	28 (15 ^c^)	1971 (but only 3 hits before 2002)
Minor attraction	“minor attract*”	21 (21)	2017
Boy/girl/child lover	“boy lover*” OR "child lover*" OR "girl lover*"	7 (5 ^d^)	1983

This search was conducted on August 31, 2021. We searched titles and author keywords.

^a^This also includes “virtuous pedophiles” (1 hit) and “non-offending/offending pedophiles” (2 hits) but excludes cases where “pedophile” is treated as an adjective (46 hits, e.g., “pedophile Internet abusers,” “pedophile community,” “pedophile priest,” “pedophile conspiracy,” “pedophile hunter,” “anti-paedophile action”) and duplicates (13).

^b^Excludes one case where the person first terminology was not applied to a different condition.

^c^Excludes 13 cases where “pedo(hebe)philic” was paired with non-people (stimuli, disorder, sexual interests).

^d^Excludes three duplicates.

### Label Preferences among People Who Are Sexually Attracted to Children

In a rare quantitative survey (Martijn et al., 2020), men who reported to be sexually attracted to children were asked to select their preferred self-labels from a list of potential alternatives. It was common for participants to endorse several options, which indicates some degree of flexibility with regard to labels. Nevertheless, the majority of the 306 participants selected the terms “child lover” (52%), followed by “pedophile” (51%), and “minor-attracted person” (40%). Other (less popular) options included “person with pedophilia” (21%), “person with hebephilia” (6%), “person with pedohebephilia” (4%), “minor-attracted adult” (12%), or “other” (7%, note that the survey did not include "pedophilic person" or similar identity first variants).

The previous qualitative literature shows that people who are sexually attracted to children struggle with the formation of a positive identity in relation to their sexuality because of its association with public stigma (Blagden et al., 2018; Muir, 2018; Nielsen et al., 2022). Only a few studies have directly investigated label preferences among people who are sexually attracted to children (Freimond, 2013; Walker, 2020). Participants in these studies expressed different attitudes toward “the P-word,” according to one participant in Freimond (2013). In Walker’s (2020) comparatively large interview study (*N* = 42, compared to *N* = 9 in Freimond, 2013), over half of the sample self-labeled as “pedophile.” However, often this was entrenched in stigma with participants quick to clarify this did not mean they were “offenders.” Some participants in Walker (2020) and Freimond (2013) stressed that the word “pedophile” needed to be reclaimed. Yet, other participants in these studies rejected the term because of concerns over being associated with “child molesters.”

Participants in Freimond (2013) and Walker (2020) also discussed alternative labels like “minor-attracted person” or “boy lover,” which some described as liberating and an improvement over terms like “pedophile.” Participants perceived the “minor-attracted person” label as accurate and less stigmatizing, but also nonspecific and, as one participant described it, “clunky” (Walker, 2020). Some preferred the more colloquial terms “boy/girl/child-lover” because they appeared to carry lesser stigma than the “pedophile” label (Walker, 2020). However, some of Walker’s study participants believed that terms like “boy lover” have negative connotations, associated with individuals who advocate for children as able to give sexual consent. Of note, Walkers and Freimond’s participants were recruited via B4U-ACT and/or Virtuous Pedophiles. Virtuous Pedophiles is a group that fundamentally opposes sexual acts between children and adults (Virtuous Pedophiles, 2022). B4U-ACT, on the other hand, prefers to focus their activism on the promotion of better mental health services at the expense of more contentious subjects like the morality of adult–child sex (B4U-ACT, 2011). Hence, it is not clear if Walkers (2020) or Freimond’s (2013) findings can be generalized to forums which do not ban or discourage such discussions in their forum, such as BoyChat (Malesky & Ennis, 2004).

### The Present Research

In the present mixed-method study, we will combine qualitative and quantitative data on label preference among people who are sexually attracted to children. Like in a previous online survey by Martijn et al. (2020), we planned to recruit a larger online sample from a variety of forums. Furthermore, the current study examined label preference in two distinct contexts (self-identity vs. use by others in professional communication) and give participants the option to indicate their level of agreement with a label (as opposed to a binary choice, as in Martijn et al., 2020) to be able to differentiate between acceptance, ambivalence/indifference, and rejection.

## Method

All materials, procedures, and analyses in this paper were pre-registered, unless otherwise stated. We uploaded the pre-registration and the study material to Open Science Framework in January 2021 before the start of the data collection (https://osf.io/ewbpa/, project https://doi.org/10.17605/OSF.IO/KPZA6). Note that we pre-registered three research questions, two of which will be featured in separate publications (Jahnke et al., 2022a, 2022b). We did not pre-register hypotheses or tests for the quantitative analyses in the present paper.

### Participants and Procedure

Data were collected between January 2021 and May 2021 on various English and German web-forums for people who are sexually attracted to children (B4U-ACT, BoyChat, Virtuous Pedophiles, VisionsofAlice, jungsforum, krumme13, kinder-im-herzen, note that we did not preregister a list of eligible web-forums). The link to our survey was furthermore shared on Twitter by members of the research team, our professional network, as well as B4U-ACT. Initially, 346 men and women completed the survey. We deleted 26 cases because participants failed the honesty or seriousness check items (see below for more information). As response time per item on a survey page was not below 1s on more than one survey page for any participant (Wood et al., 2017), we did not delete cases based on their response speed. Furthermore, we only included participants who self-reported sexual attraction to prepubescent or pubescent children that was equal to or stronger than their sexual attraction to adults, and deleted cases who indicated “no sexual attraction” to all sexual attraction items (see below for more information about measures), leading to the deletion of 34 further cases.

Hence, the final sample contained 286 pedohebephilic participants (mean age = 34.27 years, SD = 13.86, 88% male, 12% female, according to sex assigned at birth; note that 18% of the sample reported that they did not identify with their birth sex).^2^ Participants found our link on various communities (28% other website, forum or blog, 27% B4U-ACT, 15% general website, forum or blog not directed at people who are sexually attracted to children, 13% Virtuous Pedophiles, 9% BoyChat, 5% other, 3% private message, < 1% GirlChat). Their educational level was relatively high, with 85% reporting 12^th^ grade as the highest grade that they finished and got credit for. The large majority (87%) reported a dominant sexual interest in children; that is, they reported a stronger sexual attraction to prepubescent or pubescent children than to mature adults, while the remaining 13% reported an equal sexual attraction to children and adults. The sample was split about equally between people who were dominantly sexually attracted to male (42%) and female children (49%, 9% equal attraction). A slight majority reported higher attraction to prepubescent children than pubescent children (55%), while 31% reported higher attraction to pubescent than prepubescent children, and 14% reported equal attraction to prepubescent and pubescent children.

### Measures

#### Self-Reported Sexual Interests

We assessed sexual interests with a six-item scale by Jahnke and Malón (2019). Respondents indicated their sexual attraction to descriptions of the physical attributes of female and male people before, during or after puberty. These descriptions were supplemented by drawings of individuals of the respective sex and developmental stage. Note that these pictures were identical to the ones used in Schuler et al. (2021). Participants rated their sexual attraction to each of the six groups (prepubescent boy/girl, pubescent boy/girl, postpubescent man/woman) on a Likert-type scale from 1 (no sexual interest) to 10 (maximum sexual interest). We calculated scores for degree of boy attraction (maximum level of sexual attraction to a male prepubescent or pubescent child—maximum level of sexual attraction to a female prepubescent or pubescent child), and pedophilic versus hebephilic attraction (maximum level of sexual attraction to male or female prepubescent child—maximum level of sexual attraction to male or female pubescent child). Positive values on the boy attraction scores indicated dominant sexual attraction to boys, while positive values on the pedophilic versus hebephilic attraction score indicated dominant sexual attraction to prepubescent children. To assess pedohebephilia, we subtracted the maximum level of sexual attraction to adult stimuli from the maximum level of sexual attraction to prepubescent or pubescent girls or boys. Positive scores indicate a dominant sexual attraction to pubescent or prepubescent children as opposed to adults, while values of 0 indicate equal attraction to pubescent/prepubescent children and mature adults.

#### Attitudes Toward Labels

*Scale Items* Participants were asked to indicate to what degree they identified with a list of common labels for people who are sexually attracted to children (*Labeling Oneself*), and to what degree they find the use of these labels acceptable in public or academic discourse (*Being Labeled by Others*, see Table 2 for items and instructions). The labels were chosen based on what we assumed to be the most common ones based on our clinical experience, previous research on label preferences, as well as labels used by other researchers in prior research. Responses are given on a 7-point Likert-type scale with the anchors 1 (do not agree at all) and 7 (fully agree), separately for each item and for the self and other condition.

**Table 2:** Level of agreement with different labels for people who are sexually attracted to children (*N* = 285)

Item/Label	Labeling Oneself ^a^			Being Labeled by Others^b^		
	M (SD)	% agree	%unsure	M (SD)	% agree	%unsure
… a pedophile/hebephile	5.40 (2.12)	69.1	9.5	4.87 (2.27)	58.9	10.2
… a person with pedophilia/hebephilia	4.43 (2.41)	53.0	8.8	4.45 (2.40)	54.4	10.5
… a pedophilic/ hebephilic person	4.59 (2.21)	53.0	15.0	4.52 (2.22)	53.0	13.7
… a minor-attracted person (MAP)	5.63 (1.98)	76.9	8.7	5.77 (1.89)	78.7	7.0
… a boy/girl/child lover	4.83 (2.34)	58.6	9.4	3.99 (2.44)	42.5	11.9

Items ranged from 1 (do not agree) to 7 (fully agree). Missingness was negligible and ranged between 0 (no missings) and 1 (one participant refusing to answer) for each item. Note that responses above the midpoint of the scale (4) were counted as agreement, while responses on the midpoint of the scale (4) were counted as "unsure."

^a^Instruction: “Please indicate to what degree you identify with each of the following labels, in the sense that you would use them to describe yourself. I see myself as…”

^b^Instruction: “How acceptable do you find the following labels for people with a sexual interest in children in an academic or public discourse? I find it acceptable if journalists or scientists refer to a person with a

sexual interest in children as…”

*Open-ended Questions* Before participants were prompted with a list of common labels (see above), we asked the following questions: (1) “Many people use labels to describe their sexual orientation (e.g., gay, lesbian, bisexual, straight). As researchers, we are striving for a language that is inclusive and respectful. How would you label your sexual interest in children? You can also write down several labels.” and (2) “And why that particular label?” Note that the list of labels was presented on a separate page, as to not bias participants' choice of self-labels. After collecting quantitative data on attitudes toward labels, participants were asked (3) “Is there anything else you would like to add regarding labels? Please use the space below.”

#### Sociodemographic Information

We asked about sex (with response options “male” and “female”) and gender identity (with response options “male,” “female,” “something else, please specify”), age, and the highest grade in elementary school or secondary school/high school that participants finished and got credit for. We also gathered information about sexual offending status: (1) “I have been arrested, charged, or convicted for child sexual abuse,” and (2) “I have been arrested, charged, or convicted for child pornography offenses.” We also asked participants where they found the link to our survey from a list of options (“B4U-ACT,” “BoyChat,” “GirlChat,” “Virtuous Pedophiles,” “other website, forum or blog for people with a sexual interest in children not mentioned above,” “other general website, forum or blog not mentioned above [not directed at people with a sexual interest in children]”), “private message [e.g., e-mail, sms, listserv],” and “other”).

### Data Analysis

Data were analyzed using thematic analysis, a method for identifying, analyzing, and reporting patterns and themes within a data set. It aims to capture rich detail and represent the range and diversity of experience within the data (Braun & Clarke, 2006). The analysis adhered to the phases of qualitative thematic analysis as outlined by Braun and Clarke (2021). This consisted of familiarization and detailed readings of the data collected in the free text responses in the questionnaire. This then progressed to initial and systematic coding of the data and then generating initial themes from the coded data. The final phases included reviewing themes ensuring that they were consistent with the coding and that they were grounded in the qualitative data (Braun & Clarke, 2021; Smith, 2015). The final themes were representative of the sample. Taking guidance from de Wet and Erasmus (2005), Smith (2015), and Roberts et al. (2019), we also assessed a form of inter-coder agreement as a verification procedure to check coding of qualitative data. In qualitative research this occurs when two or more researchers code the exact same data independently and check for consistency across coders (de Wet & Erasmus, 2005). The second and third authors of this paper independently analyzed transcripts and then shared coding and themes in data analysis sessions with the first author to ensure that similar codes and themes were emerging from the data. The researchers met and discussed emerging themes and codes from the data, as well as both similarities and differences in data analysis. Where any differences existed, the authors discussed the different interpretations to come to a consensus regarding the interpretation of the data. As de Wet and Erasmus (2005) argue, this dialogical process can help to produce safeguards against bias, and in this study, it assisted the researchers toward inter-coder agreement.

### Honesty Check and Seriousness Check

We used the honesty and seriousness check items described in Aust et al. (2013) and Sischka et al. (2022), respectively. People indicating to have answered more than one item dishonestly or to not have been participating seriously were excluded from the research.

## Results

### Quantitative Analyses

Descriptive values for attitudes toward different labels are displayed in Table 1. On average, the label “minor-attracted person (MAP)” was rated highest, followed by “pedophile/hebephile” for both self-identification and use by others. Although “boy/girl/child lover” received relatively high endorsement rates for self-identification, it was rated least appropriate for the public or academic discourse. Both the “identity-first” and the “person-first” label received comparatively low scores. Nevertheless, although some terms were more accepted than others, “fully agree” was the response option that was most picked (i.e., the modal value) for *all* presented labels. We find high correlations (with Kendall’s τ coefficients ranging from .57 to .68) between the level of preference for the same label in the *Labeling Oneself* and the *Being Labeled by Others* condition.

Next, we tested if the average rating of each label differed significantly from the average rating of each of the remaining labels. This required conducting 20 pairwise Wilcoxon signed rank test comparisons: 10 for the *Labeling Oneself* condition and 10 for the *Being Labeled by Others* condition. We applied the Holm-adjustment to control for family-wise error rates, treating the *Labeling Oneself* condition and the *Being Labeled by Others* condition as two separate families of tests.

For *Labeling Oneself*, six of the ten mean differences were significant (all *p*s < .002): This included "pedophile/hebephile" versus the following: "a person with pedophilia/hebephilia," "a pedophilic/ hebephilic person," and "a boy/girl/child lover." Furthermore, it included the comparison of "a person with pedophilia/hebephilia" versus "minor-attracted person," and of "pedophilic/hebephilic person" versus "minor-attracted person." Lastly, we found a significant difference for "minor-attracted person" versus "boy/girl/child lover." For *Being Labeled by Others*, all 10 mean differences reached significance (all *p*s < .012), except for “person with pedophilia/hebephilia” versus “pedophilic/hebephilic person” and “person with pedophilia/hebephilia” versus “boy/girl/child lover.”

**Fig. 1:**
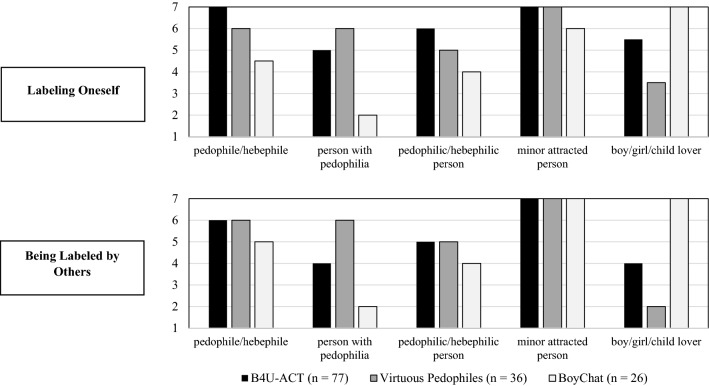
Label preferences among three selected online communities (Median). Note that the response scales ranged from 1 to 7. Higher values reflect higher identification with (Labeling Oneself) or acceptance of a label (Being Labeled by others). Note that we decided not to conduct statistical tests on these comparison because of the lack of statistical power due to small sample sizes.

Because samples sizes were low for forum subgroups, we decided to provide only a description of responses across these various forums and did not conduct significance tests. Figure 1 presents the median rating for all labels among three specific communities (B4U-ACT, Virtuous Pedophiles, and BoyChat) for *Labeling Oneself* and *Being Labeled by Others*. While “minor-attracted person” achieved high acceptance rates in all three communities, participants recruited via BoyChat also reported a strong preference for the term “boy/girl/child lover,” particular in contrast to participants from “Virtuous Pedophiles.” Participants from “BoyChat” were also less likely to accept any label that referenced “pedophilia,” with “person with pedophilia” scoring particularly low.

### Qualitative Analysis

#### Self-Chosen Labels

We will only discuss labels that were used by more than one participant and/or that could not be grouped with other labels in a meaningfully way. It was common for participants to mention several labels for themselves. A total of 195 (68%) mentioned at least one of the labels “pedophile/hebephile” or its variants, “minor-attracted person” and its variants, or “boy/girl/child lover” or its variants to refer to themselves. (Note that we had not yet introduced any of these labels at this point of the survey.) If we are also counting cases where participants used adjectives (e.g., pedophilic) or nouns referring to conditions instead of people (e.g., pedophilia), or the term “pedosexual,” this rate rises to 223 cases (or 78%). This shows that most participants produced either one or several of the terms we used in the quantitative section of the survey or a close variant (see below for a detailed description), without having been prompted by us.

*Pedophile/hebephile, person-first language, and other variants with “pedo” or “hebe” as word stems* We detected the wordstem “hebe” or “pedo” (including spelling variants “paedo” or “pädo”) in 136 individual responses (i.e., 48%, not counting two cases where participants used it in a negative sense, e.g., "anything but pedophile"). Among these 136 participants, 100 explicitly included the words “pedophile” and/or “hebephile” or a close variant or combination (e.g., pedohebephile, nepiopedophile, pedo, homopaedophile, non-offending pedophile) as a noun referring to a person. Four participants among these 136 cases used person-first language in combination with the word pedophilia (e.g., person with pedophilia or PWP). Other labels with these word stems included adjectives like pedophilic or nouns like pedophilia (without referring to a person), and, in four cases, "pedosexual."

*Minor-attracted person and variants* A total of 96 participants (34%) used the label “MAP” or “minor-attracted person” or (more rarely) a variant of the term (e.g., minor-attraction, minor-attracted man). Among this subgroup, only two participants used a version of the term that did not include the word "minor"—these participants were referring to "youth" instead. (Note that none used the word "child attracted" or "child attraction.")

*Boy/girl/child lover and variants* Fifty-two participants (18%) referred to themselves as boy, girl, or child lovers or (more rarely) used a variant of the term (e.g., "teenboylove").

*Other labels* Three participants used a more descriptive language rather than a label (e.g., "sexually attracted to little girls"), and 18 (6%) did not respond, answered "none," "whatever," that they did not know, or that they rejected labels altogether. It was also common for participants to use labels to refer to their sexual attraction to different sexes or genders to further specify their sexuality (e.g., pedohomosexual, gay/pedophile/boy lover). Of note, 26 of our 286 participants (i.e., 9%) *only* included terms to denote a sexual orientation to sexes/genders (e.g., gay, straight, heterosexual, pansexual, queer). Furthermore, three participants referenced “lolicon” or “shotacon” as labels, which refers to the erotic depiction of prepubescent or pubescent girls or boys, respectively, in Japanese manga or anime. A small number of participants self-labeled with other chronophilias (e.g., 7 mentioned nepiophilia or related terms) or other paraphilias.

### Themes

Qualitative thematic data analysis on the survey’s open text questions revealed four related themes. These were “Contested self-labels,” “Person-first language and pathologizing sexuality/identity,” “Stigma and shame,” and “Reclaiming the pedophile label.” These themes will be unpacked in the following analysis.

### Contested Self-Labels

The self-referent labels participants preferred were a matter of some contestation, and there was a theme that any label would simply become stigmatized when associated with people who are sexually attracted to children.

Extract 1

It doesn't matter what kind of labels would be given to the persons like me. We never ever would be treated with respect by any society now and, I suspect, in a distant future.

Extract 2

I don't think the word we choose is all that important. Pedophile certainly has negative connotations attached to it but I don't think MAP would help. The negative connotations would just transfer to the new word.

There was a sense of ambivalence within the qualitative data around the labels assigned and used by people who are sexually attracted to children. There was, however, some developing agreeance/support within the data that the term “minor-attracted person” (MAP) represented a more positive, less stigmatized label.

Extract 3

I think the term pedophile has a great weight, so I prefer the term MAP (Minor-attracted person) because besides not carrying misconceptions with it, it also covers other age groups of attraction.

Extract 4

"Minor-attracted person" avoids that problem, because it carries the only connotation that I am attracted to minors, not necessarily a criminal.

Extract 5

I do not know if MAP is the best label, but it is much better than pedophile. Media and the general population does not distinct well enough between persons like me that do not harm children and never has, and persons that are offenders.

While MAP was not a perfect term, participants felt it probably represented the best of the current terminology. It acknowledges their sexual interest in children, and as extract 4 highlights, it allows some measure of distancing from criminal connotations, which are perceived as common in the public discourse. However, MAP was not the only label that was prominent within this community, as “child lover,” i.e., “boy/girl lover” was a clear theme within the data and a preference for some.

Extract 6

When I'm chatting with online friends about life in general, I like to use the term Boy Lover since that is what I am at heart.

Extract 7

Girl Lover is my preferred label because it expresses my enthusiastic passion for little girls!

Extract 8

I like boy lover because it is common in my groups and it communicates love.

Extracts 6–8 demonstrate how the “boy/girl” lover label has support and endorsement from those within the MAP community, particularly as this label was perceived to convey feelings of love and romance and not just sexual interest. The data points to a distinction between public and private identities, as while publicly participants would not define themselves as, e.g., “boy lover,” privately and within the communities they belong such labels were important.

Extract 9

And while "boylover" is probably the most accurate [label] it's one of those terms really only used with other boylovers.

Extract 10

I personally resisted BoyLover for a time, because it seems on the surface to be "someone who is a lover to boys" rather than "someone who loves boys". The former is clearly sexual while the latter is more romantic

As extract 9 highlights that while privately “boylover” may be internalized or be construed as “accurate,” the label is only used in the private sphere. Extract 10 further highlights tensions between labels used within MAP communities and points to resistance to the label due to implied sexual connotations.

#### Person-First Language and Pathologizing Sexuality/Identity

In past decades, professional bodies like the APA have promoted the use of person-first language to increase acceptance of those who are carrying a specific label. However, this was disputed within the data as “person with” language was construed as promoting “othering” or increasing stigma in some instances.

Extract 11

I dislike “a person with pedophilia.” It makes it sound like an illness, which I don't believe it is.

Extract 12

I feel like saying "a person with pedophilia" makes it sound like a sickness or something affecting the person.

Extract 13

However, "a person with ..." sounds a bit like a person with a (medical) condition. I don't see pedophilia/hebephilia as a medical condition, but as a sexual orientation.

The qualitative data demonstrated that participants strongly felt that some person-first language instead of reducing stigma, actually served to increase it as it could be perceived as a “sickness” or “illness.” Some also articulated that such labels made individuals feel worse as it was pathologizing a core aspect of who they are.

Extract 14

There's also some debate on referring to it as a sexuality, disorder, or mental illness.

Well we should start to use labels that help people like me instead of making us feel shitty.

Extract 15

“A person with pedophilia" sounds like I have a disease and seems to imply I can be cured somehow. I know there is controversy around the idea of pedo/hebephilia as an orientation but "a person with homosexuality" sounds ridiculous because it is. It's not additional to who I am. It's part of me.

While person-first language is construed as more respectful and separates the person from a behavior, researchers/practitioners need to be mindful of the limits to this and understand the instances where it may be perceived as stigmatizing.

#### Stigma and Shame

Linked with divergences within the data regarding “person-first” language and labels, identity struggles due to shame and stigma was a further important theme. Shame is a useful concept when understanding private selves, as it is a discrepancy between what the person wants to be or how they identity and the way that person is being identified socially (Lazarus, 2006). This tension and the experience of stigma were prominent within the qualitative data.

Extract 16

There is such a strong stigma against the word pedophile outside of the MAP community that using that can automatically garner negative reactions from the public.

Extract 17

The general public struggles in understanding what terms relating to pedophilic sexual preference actually mean, so journalists should avoid using the term "pedophile," as people often interpret it to mean "child molester."

Extract 18

The label "pedophile" is tainted and confused with abuser so I hate it.

Most participants believed terms like “pedophile” were shame and stigma inducing that they were “tainted” and that being associated with such labels meant they shared that stigma.

Extract 19

The word "pedophile" is now seen as synonymous with "child molester". For me, the two terms are very different, but society doesn't see it that way.

Extract 20

I am attracted to minors, yet I do not act on my feelings and as such shouldn't be branded as a monster like pedophiles are.

Individuals with minor-attraction do not live in hermetically sealed vacuums and are aware of the stigma and shame associated with such labels. Extract 20 demonstrates participants’ views that such labels are like a form of branding. There was also the recognition from participants that “pedophile” was construed as a “child molester” by the public.

#### Reclaiming Pedophile Label

Despite a strong theme around the stigma associated with labels like “pedophile,” there was a theme from some of the participants in terms of attempting to reconstruct and reclaim the label “pedophile.”

Extract 21

I think we have to ignore the insult and just reclaim pedophile as a colloquial word for a neither good nor bad sexual orientation [but] that would include all shades of minor-attraction... Trying to distance from the pedophile label suggests that there's something wrong with being a pedophile.

While distancing from labels such as “pedophile” may have adaptive benefits for an individual’s identity, some believed that such distancing simply reinforced the stigma for the rest of the community.

Extract 22

A pedophile is what I am and there isn't any flowery language to deflect that.

Extract 23

I like to be straight and to the point. Pedophilia is a sexual interest in prepubescent children. I understand the negative connotations some people associate with the term, but I don't, and I won't change what I call myself just because some people are underinformed. I'll gladly explain what I mean if asked, but I am a pedophile, and no one can take that term from me.

A small of number of participants identified with the label “pedophile” and wanted to take ownership of the term. They felt the label best reflected their sexual interest and their personal identity (“It’s who I am”), irrespective of what others may think about it.

## Discussion

The purpose of the current study was to investigate the label preferences of people who are sexually attracted to children. Quantitative analysis showed overall acceptance of the five labels “pedophile/hebephile,” “person with pedophilia/hebephilia,” “pedophilic/hebephilic person,” “minor-attracted person,” and “boy/girl/child lover.” Some labels were, however, less preferred than others (see below for a more detailed discussion). There appear to be differences between different online communities of people who are sexually attracted to children: while the term “minor-attracted person” was accepted by most participants across groups, “person with pedophilia” and “boy/girl/child lover” appeared to be considerably more divisive. Moreover, we found little difference between the preference for different terms between self-labeling contexts and professional contexts, where one would be labeled by others. This means that someone who accepts a label for the self would also be highly likely to condone the use of the same label by others.

Qualitative analysis uncovered four themes (“Contested self-labels,” “Person-first language and pathologizing sexuality/identity,” “Stigma and shame,” and “Reclaiming the pedophile label”). Besides giving an insight into the sense making and reasons for accepting, reframing, reclaiming, or rejecting the aforementioned labels, the themes highlight the difficulty associated with finding a non-stigmatizing label and balancing public and private identities (Goffman, 1963). In contrast to a recent review that finds both positive and negative aspects of diagnostic labels on self-concept (O’Connor et al., 2018), participants in our survey mostly described labels as a threat to their self-esteem and the development of a positive identity.

Similar to findings in Martijn et al. (2020), “minor-attracted person” and “pedophile/hebephile” emerged as popular labels in our survey, with participants appreciating the distinction from sexual offending and other presumably more contentious labels like “pedophile.” Both labels were rated as more acceptable across both the “Self-Label” and the “*Being Labeled by Others*” condition compared to the three other options. Note that the higher relative popularity of the term “child lover” in Martijn et al. (2020) compared to the present study may be due to Martijn et al.’s focus on romantic attraction to children (while ours was advertised as a study on labels, attitudes toward therapy, and experiences in therapy). Hence, people who self-label as “child lover” may have been more likely to self-select to Martijn et al.’s study than ours.

Echoing the debate around the use of person-first terminology in disability research (Dunn & Andrews, 2015; Gernsbacher, 2017; Mackelprang, 2010), person-first labels were the target of much criticism because of their implication that pedophilia is undesirable, unwanted, and innately pathological (see also Martijn et al., 2020 where this was among the least popular self-labels). Both qualitative and quantitative findings suggest that people who are sexually attracted to children prefer to embrace their sexuality as part of their identity and want this to be reflected in the professional discourse as well. When participants were given the option to freely state which label they preferred, only a very small minority used person-first language. Interestingly, although participants rarely commented on the use of identity-first labels, this alternative to person-first language was not perceived as a preferable option to the latter in our quantitative analysis. However, not that this observation only holds true for a narrow conception of identity-first language, whereby the personhood noun is combined with a clinical term (i.e., "pedophilic person"), as the nonclinical label "minor-attracted person" was undeniably popular. Our data furthermore indicate (on a descriptive level) that people who frequent BoyChat, as one of the forums that do not discourage or ban forum posts that condone adult–child sex, are more likely to reject clinical terms like “pedophilia” or “hebephilia” for their sexual attraction than users of other forums. However, larger datasets (potentially built on analysis of forum messages) are needed to determine the robustness and significance of these effects.

While our quantitative data indicated few distinctions between public and private use of labels, we noted a discrepancy between the label “boy/girl/child lover” for oneself as opposed to accepting its use by others. Note that this label was also the only label that was not endorsed in a public or academic context by most participants. The qualitative data indicate that one reason for this may be the word’s potential criminal connotations (which is also discussed by participants in Walker, 2020). However, the term “boy/girl/child lover” emerged as a popular label among the subgroup of BoyChat users. Note that this does not necessarily mean that people who endorse this label or who use BoyChat are in favor of adults engaging in sex with children. In fact, our qualitative data indicate that some understand this term to mean "someone who loves children" rather than "someone who is a lover to children."

A few participants expressed the fatalistic view that all labels associated with their sexual attraction would become tainted. These fears are not unfounded, as public reactions to those who are sexually attracted to children are extremely negative, even when contrasted with other paraphilias, alcohol abuse, or antisocial traits, and even when the person in question is described as non-offending (Jahnke et al., 2015a, 2015b; Lehmann et al., 2020). This stands in contrast to hopes that alternative labels could decrease the public shame associated with sexual attraction to children. For instance, there is evidence that interventions that educate about the distinction between a sexual attraction to children and sexual offending lead to a decrease in stigmatizing attitudes among students, health professionals, or members of the general public (Harper et al., 2022; Heron et al., 2021; Jahnke et al., 2015a, 2015b; cf. Jara & Jeglic, 2021 who report a stigma-increasing effect). Therefore, by shedding the criminal connotations of the label “pedophilia,” labels like “minor-attracted person” could help the public understand the difference between having a sexual attraction toward children and committing sexual crimes with child victims. Nevertheless, and in keeping with the more pessimistic tone of some respondents, a debate about language change regarding such a highly emotive topic can quickly lead to outrage and threats of violence, as in a recent high-profile case involving a fellow academic (documented in Letourneau & Malone, 2021). In this case, speaking in favor of the label "minor-attraction" drew intense public condemnation, fueled by the quick and incorrect assumption that the researcher was attempting to normalize sexual offending against children (Bailey, 2022).

Some participants in this study expressed views in favor of reappropriating the “pedophilia” label as a way to express ownership, pride, and agency in the face of a misinformed public. Note that this idea is also discussed by participants in Freimond (2013) and Walker (2020). There is nascent research on the effects of reclaiming a slur among self-labelers and observers (Galinsky et al., 2013). These studies suggest that self-labeling with a slur can lead to an increased sense of power among the self-labeled. Additionally, when others observed that members of a stigmatized group used a slur to refer to themselves, they became more likely to view the self-labeler as powerful. Galinsky et al. (2013) also demonstrated that reclaiming a slur can attenuate the negativity of the label via increased perceptions of the group’s power. Yet, it is unclear if the ramifications of self-labeling as a “pedophile” would produce similar results among the general public. Given the fact that the stigma attached to pedophilia is so severe and interwoven with concerns about child protection, self-labeling as a "pedophile" in a way that expresses confidence and pride may prompt violent reactions. Research is needed to explore public reactions to various labels for people who are sexually attracted to children, for instance, by analyzing public reactions to social media posts where people who are sexually attracted to children discuss their old or new identities.

### Limitations

As with other online studies, results are limited by an unrepresentative sample of participants, which over-represents participants with a high level of education and, possibly, a range of other variables (e.g., interest in research and activism, agreeableness). Notably, when compared to US national rates, an unexpectedly large number of participants in this survey reported to not identify with their gender assigned at birth, particularly people who were born female. Yet, this does not appear to be atypical for online community samples (Stephens & McPhail, 2021). It is also likely that the current study over-represents participants with a “no contact” or at least a critical stance with regard to the moral permissibility of sex between adults and children. This is because these groups are likely to perceive a stronger alignment between their own moral attitudes and goals and those of the research team, especially since the main content of the survey was on attitudes toward and experiences with treatment. This is relevant for the generalization of our results, as it indicates that preferred terminologies are tied to specific online communities. People who seek online forums may also be more exclusive and stable in their sexual attraction to children than those who do not, which makes it more likely to become a core aspect of their identity. The process of engaging with others is also likely to help users establish a positive self-image and to distance themselves from stigmatizing societal perceptions (Holt et al., 2010; Nielsen et al., 2022). This process may lead to increased identification with the group label and rejection of labels with medical/pathological connotations (such as identity-first or person-first language). Hence, it is possible that people who are sexually attracted to children but *not* involved in such online communities might hold different attitudes toward the herein presented labels. For that reason, it would be of interest to research attitudes toward labels among, for instance, clinical or forensic samples of people who are sexually attracted to children.

Our study is furthermore limited by its focus on a select few labels among many (and potentially endless) other options. Nevertheless, the majority of the participants reported at least one label for themselves that corresponded or at least closely corresponded with the ones that we had listed. (Note that this particular open-ended question prefaced our list of labels, which was presented on a separate page.) There was no term in our reading of alternative labels that stood out by virtue of being favored by more than ten participants, referencing sexual attraction to children (as opposed to other types of attraction to, for instance, different sexes or genders), *and* differing substantially from the labels that we had listed in our study materials. This gives us confidence in our decision to focus on these particular labels in the quantitative section of our survey was reasonable. Future researchers may nevertheless consider including labels for participants attracted to small children (i.e., nepiophilia) or, particularly in the *Being Labeled by Others* condition, longer and more descriptive formulations like the one adopted within this very paper ("people who are sexually attracted to children”).

Furthermore, we tested only one version of person-first and identity-first terminology (i.e., "person with pedophilia/hebephilia" or "pedophilic/hebephilic person") instead of other potential personhood nouns like "man/woman," "someone," or "(an) individual." We are not aware of prior research indicating differential effects for different personhood nouns in the context of bias-free medical language, and the qualitative data did not indicate that participants would have preferred another personhood noun over the one that we included. This does not rule out the possibility that other formulations would have been received differently. Nevertheless, we also do not subscribe to the belief that subjecting participants to rate a myriad of potential labels, which differ in nuances, constitutes a more fruitful approach. Instead, we think that more can be learned from understanding the dimensions underlying the rejection and adoption of various labels. For most in our community sample, their label preferences appear to be guided by a desire to have a label reflect that their sexual attraction is not pathological or detached from their identities, and that is not confused with child sexual offending.

Even though we employed several quality checks, the anonymous nature of the study necessitates a reliance on self-report, which may be faked or inaccurate. However, at least with regard to sexual attraction patterns, previous research shows a high correlation between self-report and indirect measures among online samples of people who are sexually attracted to children (Jahnke et al. 2022c). Lastly, since labels are context-specific, it is unclear how translated versions of the presented labels (or similar terms) would be received by participants who do not speak English.

### Implications for Research on People Who Are Sexually Attracted to Children

For better or worse, professional bodies like the American Psychological Association (2019) increasingly expect researchers to keep track of the labels that are preferred within and by the communities that they want to study. The present study confirms previous findings that attitudes and preferences of people who are sexually attracted to children vis-a-vis various labels are not homogenous (Martijn et al., 2020; Walker, 2020). Person-first language and terms like “boy/girl/child lover” are particularly contested and may alienate some subgroups of people who are sexually attracted to children. Nevertheless, and bearing in mind the limitations of the current study, the use of the label “minor-attracted person” appears to be a safe option of addressing people who are sexually attracted children and has the added benefit of differentiating between sexual attraction and criminal behavior. Nonetheless, scientists need to be aware of potential downsides of using new terminology, such as impeding efficient and precise communication, and creating challenges to connect past, present, and future research (Bailey, 2019). The term minor-attraction obscures, to some extent, the attraction at stake—if we assume that being attracted to postpubescent minors (i.e., between 15 and 17; Seto, 2017) is normal for teleiophilic individuals, most people could be deemed MAPs. Therefore, researchers who are using this label need to be very clear about the age or sexual maturity ranges that would be covered by the term, both when addressing professional audiences, the general public, and people who are sexually attracted to children.

## Footnotes

1 In the present article, we choose to assume a neutral position with regard to our interest of study, by referring to this group descriptively as "people who are sexually attracted to children" throughout. Note that this is also the term we used in our recruitment and study materials to minimize the risk of selection bias with regard to people with specific label preferences.

2 This is a noteworthy finding, as a recent representative poll shows that less than 1% of US-Americans identify as transgender (< 2% among people aged 18 to 22 years; Gallup Inc., 2021). Among the 51 transgender participants, 15 were transmen (female-to-male) and 8 were transwomen (male-to-female). Among the 28 who indicated that they identified as “something else,” 8 reported their birth sex as “female.” That means that while this sample included 35 people with female birth sex, the majority of this group (i.e., 66%) did not report “female” as their gender identity.
